# Global Trends in Research of Macrophages Associated With Acute Lung Injury Over Past 10 Years: A Bibliometric Analysis

**DOI:** 10.3389/fimmu.2021.669539

**Published:** 2021-05-20

**Authors:** Sheng Wang, Huanping Zhou, Li Zheng, Wanli Zhu, Lina Zhu, Di Feng, Juan Wei, Guannan Chen, Xiaohong Jin, Hao Yang, Xuan Shi, Xin Lv

**Affiliations:** ^1^ Department of Anesthesiology, Shanghai Pulmonary Hospital, School of Medicine, Tongji University, Shanghai, China; ^2^ Department of Anesthesiology, Fuyang Hospital of Anhui Medical University, Fuyang, China; ^3^ Department of General Surgery, Shanghai Pulmonary Hospital, School of Medicine, Tongji University, Shanghai, China; ^4^ Department of Anesthesiology, The Second Affiliated Hospital of Nanchang University, Nanchang, China

**Keywords:** bibliometrics, acute lung injury, macrophages, bibliometrix, VOSviewer

## Abstract

Acute lung injury (ALI) is an intractable disorder associated with macrophages. This bibliometric analysis was applied to identify the characteristics of global scientific output, the hotspots, and frontiers about macrophages in ALI over the past 10 years. We retrieved publications published from 2011 to 2020 and their recorded information from Science Citation Index Expanded (SCI-expanded) of Web of Science Core Collection (WoSCC). Bibliometrix package was used to analyze bibliometric indicators, and the VOSviewer was used to visualize the trend and hotspots of researches on macrophages in ALI. Altogether, 2,632 original articles were reviewed, and the results showed that the annual number of publications (Np) concerning the role of macrophages in ALI kept increasing over the past 10 years. China produced the most papers, the number of citations (Nc) and H-index of the USA ranked first. Shanghai Jiaotong University and INT IMMUNOPHARMACOL were the most prolific affiliation and journal, respectively. Papers published by Matute-Bello G in 2011 had the highest local citation score (LCS). Recently, the keywords “NLRP3” and “extracellular vesicles” appeared most frequently. Besides, researches on COVID-19–induced ALI related to macrophages seemed to be the hotspot recently. This bibliometric study revealed that publications related to macrophages in ALI tend to increase continuously. China was a big producer and the USA was an influential country in this field. Most studies were mainly centered on basic researches in the past decade, and pathways associated with the regulatory role of macrophages in inhibiting and attenuating ALI have become the focus of attention in more recent studies. What is more, our bibliometric analysis showed that macrophages play an important role in COVID-19–induced ALI and may be a target for the treatment of COVID-19.

## Introduction

Acute lung injury (ALI), leading to acute respiratory distress syndrome (ARDS), is a common critical disease resulting from sepsis, pneumonia, trauma, acute pancreatitis, and inhalation of gastric contents. Among them, sepsis is the leading cause. The main pathophysiology of ALI is characterized by diffuse alveolar injury, pulmonary edema, and excessive inflammatory response (1, 2). Macrophages, with considerable diversity and plasticity, are the key to maintaining lung immune homeostasis. In response to various signals (such as microorganisms, damaged tissues and activated lymphocytes), macrophages may polarize into classical M1 or alternative M2 with different functional phenotypes (3). Both of them play a central role in the development and progression of ALI (4). Consequently, it is significant to quantitatively analyze the status quo, focus areas, and future prospects related to macrophages in ALI.

Bibliometrics is an area of studying library and information science by analyzing the bibliographic material using a quantitative measure (5). As a convenient method, bibliometrics can estimate the developmental trend in a scientific file and reveal the key research directions through analyzing databases and characteristics of the literature. In addition, it can provide effective proof for guiding experimentation strategies and funding decisions (6). Over the years, fruits of bibliometric analysis have been applied to the medical fields of gynecology, orthopedics, complementary, and alternative medicine (7–9). However, bibliometric study on macrophages in ALI remains a void. Thus, the aim of the present study was to systematically analyze the research of macrophages in ALI to evaluate the current state and hotspots in this field.

## Materials and Methods

### Data Sources and Search Strategies

**Figure 1:**
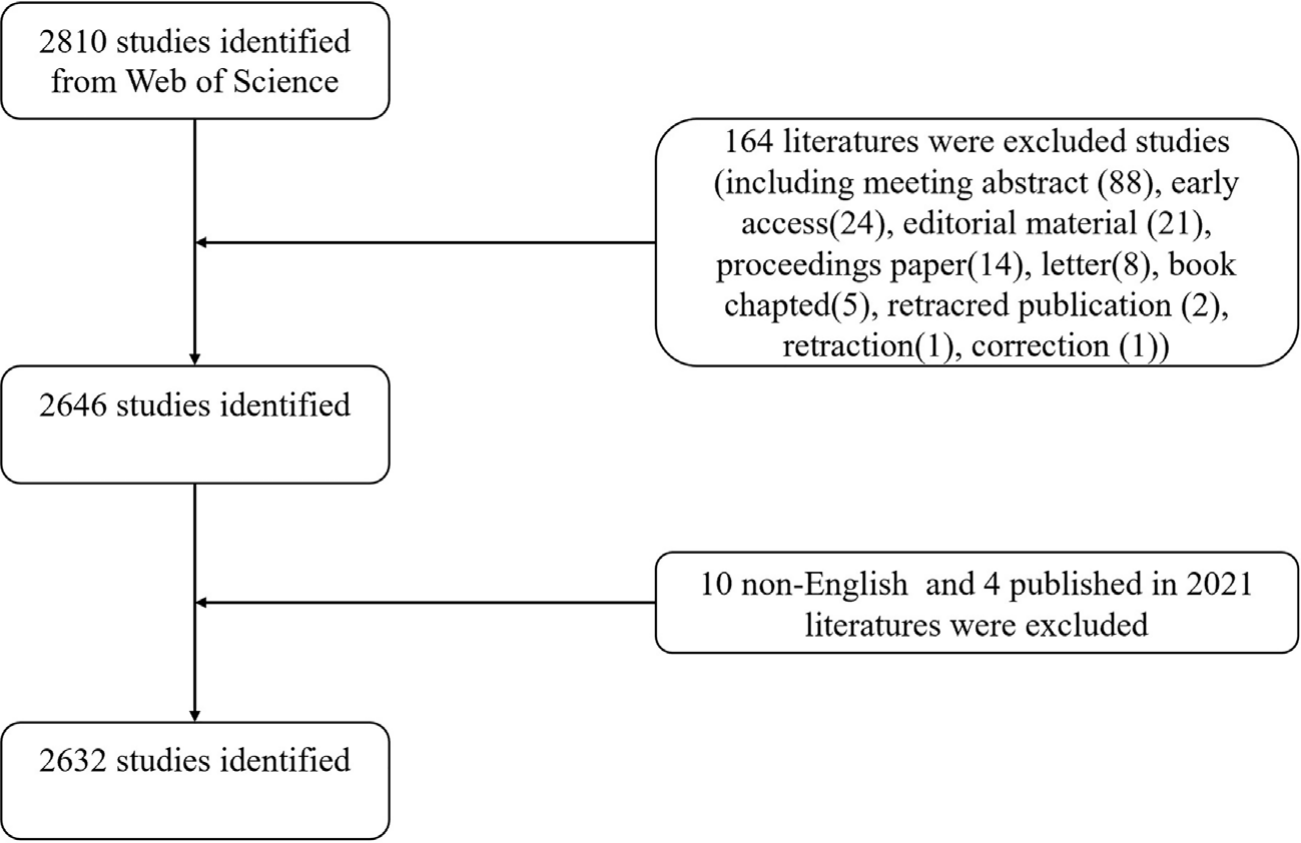
Flowchart of the screening process.

We used the SCI-expanded of WoSCC bibliographic database developed by Thomson Scientific to perform bibliometric analysis. Considering rapid database renewal, literature retrieval was conducted on a single day (January 17, 2021) to avoid deviations. The publication period in this study was set between 2011 and 2020. The search terms were presented as follows: TS= (macrophage OR macrophages) AND TS= ((acute lung injury) OR (acute respiratory distress syndrome) OR (ALI) OR (ARDS) OR (respiratory distress syndrome)). Of various publication types, only original articles and reviews written in English were included. Two researchers (SW and HZ) independently conducted the primary data search and then discussing any potential differences, the final agreement reached 0.90, showing substantial accordance (10). In total, 2,632 articles were ultimately analyzed in our study. The detailed screening is shown in **Figure 1**.

### Data Collection and Cleaning

Firstly, the original data were extracted from the SCI-expanded database. The recorded information included the number of papers and citations, H-index, publication year, countries/regions, affiliations, authors, journal, references, and keywords. Afterward, duplicate authors and misspelled elements were removed artificially. Although inaccurate analysis may not be avoided completely due to multiple versions of cited references, the same abbreviated name of different authors, and different forms of cited journals, we believe that most raw data were reliable. Before data analysis by VOSviewer v.1.6.15.0 (Centre for Science and Technology Studies, Leiden University, Leiden, the Netherlands), a thesaurus file was used to merge some duplicates into one word, correct the misspelled elements and delete the useless words. Finally, the cleansed data were imported into Bibliometrix package and VOSviewer for bibliometric analysis.

### Bibliometric Analysis

Bibliometric indicators included the number of papers and citations that were frequently used to represent the bibliographic material. Generally, the number of publications (Np) was used to measure productivity, and the number of citations without self-citations (Nc) was used to represent the impact, knowing that they are two main perspectives to evaluate the level of research. More recently, H-index has been increasingly used to evaluate the academic contribution of a researcher and predict future scientific achievements (11, 12). H-index unifies productivity and impact by finding the threshold that connects Np with Nc. In other words, if a researcher published H papers and each of the papers at least had been cited H times, she or he would have an H-index (13). Particularly, although H-index was initially developed to evaluate individual academic achievement, it could be extended to describe the publication output of a nation or region, an institution, or a journal (14). Besides, the impact factor (IF) obtained from the latest version of Journal Citation Reports (JCR) has been widely viewed as one of the leading measures of quality and impact of medical journals (15). As an important indication of contribution, local citation score (LCS) is regarded as the Nc of an article inside one specific field. To some extent, it can indicate the degree of innovation of an article in the knowledge domain (16).

R (Version 4.0.2) is the language and environment for statistical computing and graphics. It is highly extensible and can be used to automate the analyses and create new functions. Bibliometrix package in R was used to perform a basic bibliometric analysis of the cleansed data (17) and analyze LCS. To further illustrate the changes in the annual document quantity, the fitting polynomial model was applied to predict the annual Np. Variable f (x) represents the annual number of studies, and x denotes the publication year.

Additionally, the study also constructed bibliometric maps *via* VOSviewer software to obtain more comprehensive information of the result based on co-citation and co-occurrence (18). Co-citation was defined when two items were both cited by the third item. Co-occurrence of keywords measures the most frequent keywords that appear in the same documents (19), knowing that analysis of co-citation references and co-occurrence keywords can illustrate the research hotspots related to macrophages in ALI.

## Results

### An Overview of Publications on Macrophage in ALI

Based on the search strategy, a total of 2,632 articles and reviews published in the past decade were retrieved. The total Nc for the retrieved articles was 57,256, and the mean Nc per article was 21.75. The H-index of all publications was 88.

### The Annual Trend of Paper Publication Quantity

**Figure 2:**
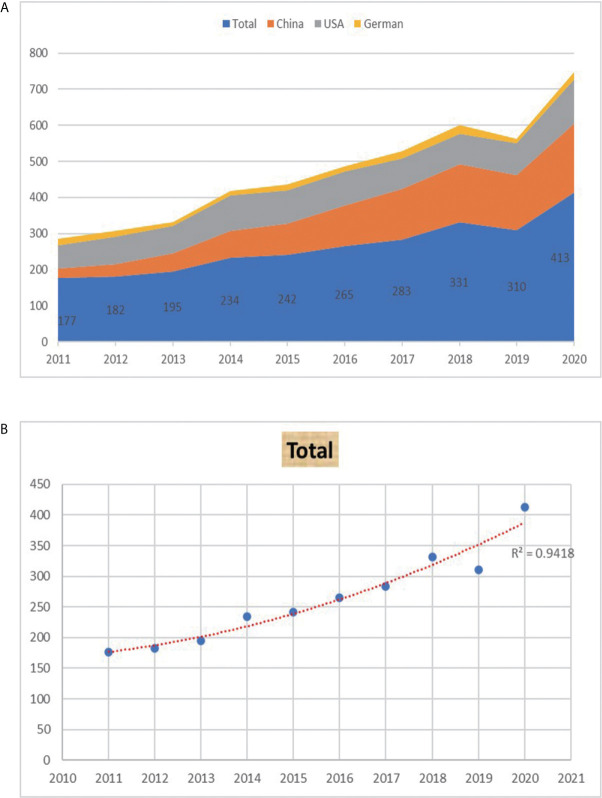
**(A)** The number of publications by year over the past 10 years. **(B)** Curve fitting of the of the total annual growth trend of publications (R^2^ = 0.9418).

**Figure 2A** shows the annual Np related to macrophages in ALI. In general, despite fluctuation in 10 years, the number of annual papers rose from 177 in 2011 to 413 in 2020, and the Np grew to the peak in 2020. From 2011, the annual Np in the USA and Japan remained stable, while it increased rapidly in China. **Figure 2B** illustrates the polynomial-fitting curve of the annual trend of paper publication quantity. The annual Np was significantly correlated with the publication year, and the correlation coefficient R^2^ reached 0.9418 according to **Figure 2B**. Overall, these findings indicate that research on macrophages in ALI has become the focus of attention and entered a stage of rapid development.

### Contributions of Countries/Regions to Global Publications

We ranked 10 high-output countries/regions of all authors according to the Np (**Table 1**). China published the most articles (1,026/38.98%), followed by the USA (878/33.36%) and Germany (162/6.16%). Papers from the USA were cited by 23,791 times, accounting for 41.55%. of the total citations, followed by China (15,336) and England (3,421). In addition, the USA achieved the highest H-index (73), more than twice the figure for Germany (32). Compared with Japan and China Taiwan, the NP in UK and Canada was moderately lower but with a remarkably higher H-index and Nc.

**Table 1:** Publications in the 10 most productive countries/regions.

Rank	Country/Region	Np	% of (3,575)	Nc	H-index
1	China	1,026	38.98	15,336	51
2	USA	878	33.36	23,791	73
3	Germany	162	6.16	3,378	32
4	Japan	136	5.17	2,202	25
5	South Korea	115	4.37	2,408	28
6	China Taiwan	104	3.95	1,381	21
7	Canada	100	3.80	2,662	27
8	England	95	2.62	3,421	31
9	Brazil	75	2.85	1,627	18
10	France	72	2.74	1,731	23

### Analysis of Affiliations

**Table 2** displays the top 10 affiliations with the highest number of publications related to macrophage in ALI. Shanghai Jiaotong University had the highest Np (82), followed by Fudan University (72) and Zhejiang University (53). Fudan University ranked first in terms of the Nc (1,579), and Jilin University had the highest H-index. Although the Np of Wenzhou University and Tongji University in China was relatively high, their H-index lagged far behind other productive universities. China was home to most affiliations, excluding schools University Michigan and University Colorado in the USA and University Toronto in Canada.

**Table 2:** The top 10 productive affiliations.

Rank	Affiliations	Country	Np	Nc	H-index
1	SHANGHAI JIAOTONG UNIV	China	82	1,282	18
2	FUDAN UNIV	China	72	1,579	22
3	ZHEJIANG UNIV	China	53	1,335	15
4	WENZHOU UNIV	China	52	545	14
5	UNIV MICHIGAN	USA	51	1,436	22
6	JILIN UNIV	China	45	1,256	23
7	TONGJI UNIV	China	44	572	14
8	HUAZHONG UNIV SCI TECHNOL	China	43	716	15
9	UNIV COLORADO	USA	41	920	16
9	UNIV TORONTO	Canada	41	1,339	20

### Analysis of Authors

The top 10 productive authors are listed in **Table 3**. They contributed 177 publications, accounting for 6.72% of the total number of papers. Liang, G from the Wenzhou Medical University tied for the first place in the field of investigating macrophages in ALI, followed by Zhang, YL from Wenzhou Medical University in China and Laskin, D. L from Rutgers State University in the USA. As shown in **Table 3**, Matthay, M. A had a remarkably high Nc. In addition, most of the top 10 authors were from the USA (5) or China (4).

**Table 3:** The top 10 authors with the most publications.

Rank	Author	Country	Np	Nc	H-index
1	Liang, G.	China	24	326	10
2	Zhang, YL.	China	19	275	10
2	Laskin, D. L.	USA	19	377	12
4	Wang, P.	USA	18	234	9
5	Matthay, M. A.	USA	18	2,021	12
6	Laskin, J. D.	USA	17	310	10
6	Thorlacius, H.	Sweden	17	326	10
8	Gow, A. J.	USA	15	231	8
8	Sun, L.	China	15	197	8
8	Qian, F.	China	15	137	8

### Analysis of Journals

As presented in **Table 4**, the International Immunopharmacology (97 publications, IF: 3.943) published the most papers concerning macrophages in ALI, and the American Journal of Physiology—Lung Cellular and Molecular and Physiology (87 publications, IF: 4.406) and the Plos One (85 publications, IF: 2.740) ranked the second and third respectively. Approximately 25% of the papers were published in the top 10 academic journals (642/24.4%). Among the top 10 journals, apart from Plos One (IF: 2.740), Shock (IF: 2.960) and the Journal of Surgical Research (IF: 1.841), the rest were journals with high IF (defined as greater than 3.000). Notably, the American Journal of Respiratory Cell And Molecular Biology (IF = 5.373) has higher citations and H-index.

**Table 4:** The top 10 most active journals.

Rank	Journal	Np	H-index	Nc	IF (2019)
1	INT IMMUNOPHARMACOL	97	24	1,685	3.943
2	AM J PHYSIOL-LUNG C	87	28	2,720	4.406
3	PLOS ONE	85	22	1,699	2.740
4	J IMMUNOL	69	29	2,195	4.886
5	FRONT IMMUNOL	58	14	901	5.082
6	INFLAMMATION	55	84	967	3.212
7	AM J RESP CELL MOL	54	24	2,475	5.373
8	SHOCK	52	16	841	2.960
9	SCI REP-UK	49	16	862	3.998
10	J SURG RES	36	15	677	1.841

### Analysis of Paper Local Citations (LCS)

**Figure 3:**
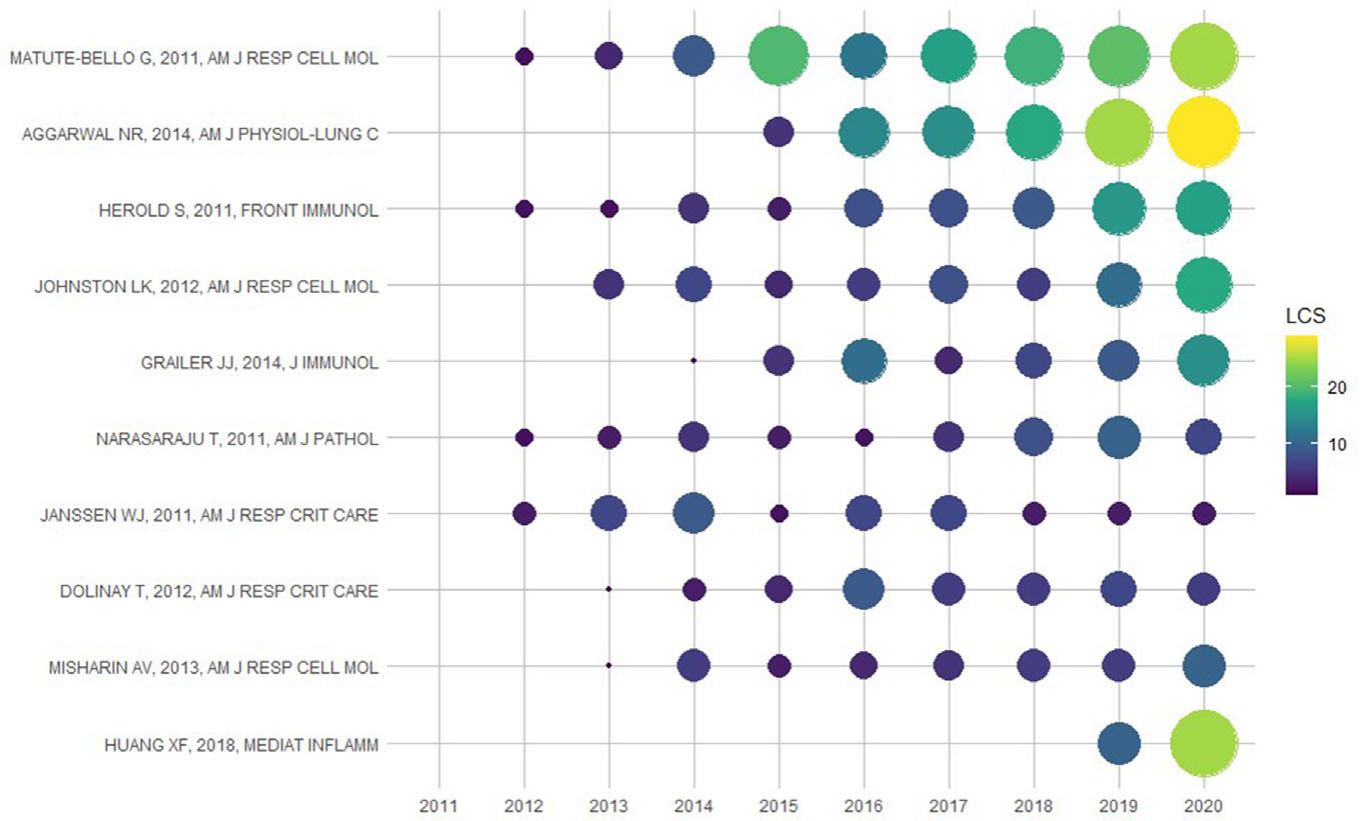
The yearly number of local citations of papers with high local citations (LCS). The size and colors of the circle represent the LCS of papers.

The number of LCS per year for the top 10 articles is presented in **Figure 3**. The LCS of the paper written by Matute-Bello, G in 2011 was 129, ranking the first. In this paper, the authors summarized the main features characterizing ALI in animals and identified the most relevant methods of assessing these features (20). In addition, the works of Aggarwal et al. (4); Herold et al. (21); Johnston et al. (22); and Huang et al. (23), which pointed the relationship between diverse pulmonary macrophage subpopulations and different phases of ALI won more LCS in recent years. Meanwhile, Grailer et al.’s (24) work of NLRP3 inflammasome activating macrophages in ALI had higher and higher LCS, while Janssen et al.’s (25) work about apoptosis of recruited macrophages in ALI was the opposite. Anyway, these documents made a difference in studying macrophages in ALI and could be described as seminal, which increased the number of subsequent literature publications in this field.

### Analysis of Co-Cited References

**Figure 4:**
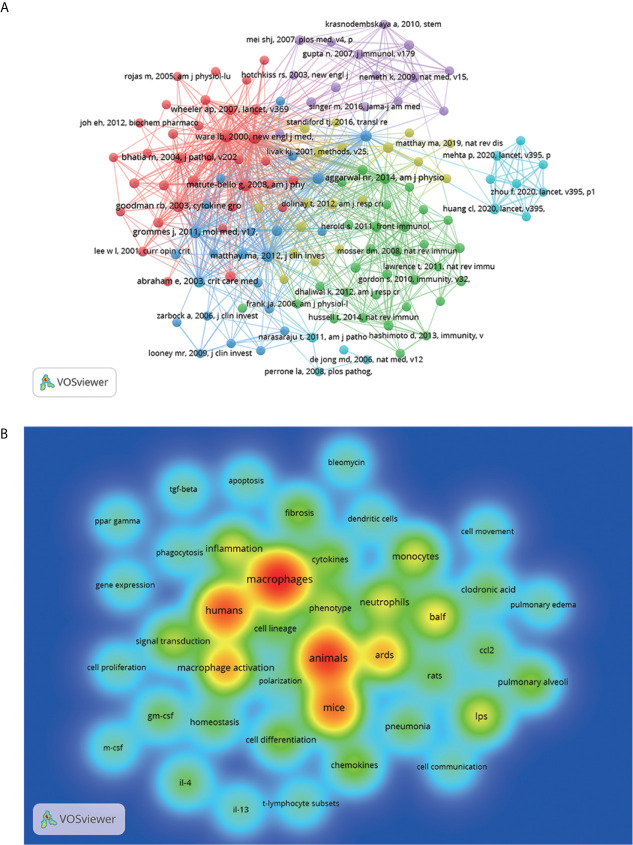
Mapping on co-cited references of studies related to macrophages in ALI(25 citations). **(A)** Network map of co-cited references. Of the 89,312 references, 133 (classified into six clusters) had at least 25 times cited. **(B)** Density visualization for keywords of cluster2 in co-cited references network map. Each keyword in the density visualization has colors that indicates its density. Red means appearing more frequently, while green means appearing less frequently.

Unlike local citation analysis, the co-citation network lays stress on the research themes closely related to a specific field (26). Considering masses of cited references, the minimum number of citations of a reference was set as 25. Of the 89,312 references cited by the retrieved papers, 133 were selected for co-citation analysis (**Figure 4A**). The line between two nodes means that both were cited in one publication and a shorter line represents a closer relationship between two papers. The size of nodes represents the total link strength, representing the total number of co-citations of a document. In addition, different colors of nodes were used to divide the papers into different clusters. Cluster 1 (in red) included 34 references, which mainly focused on the mechanism and pathway of the inflammatory stage of ALI. Cluster 2 (in green) mainly paid attention to the regulatory effects of diverse macrophage populations on ALI. Cluster 3 (in blue) centered on clinical characteristics and animal models of ALI. The theme of cluster 4 (in yellow) is the critical role of Inflammasome in ALI development. Cluster 5 (in purple) focused on the treatment of stem cells in ALI caused by sepsis. Cluster 6 (in cyan), which was mostly about the relationship between Covid-19 and ALI, was far away from other clusters. It suggested cluster 6 grew so rapidly in a short period of time and had not yet established a close relationship with other clusters.

For further study of the co-citation of macrophage function-related references, density visualization was used to analyze 31 references in cluster 2 (**Figure 4B**, **Supplementary Table 1**). Density visualization often shows the overall structure of the study and highlights important research areas (27). **Figure 4B** showed that macrophage functions in ALI were the theme of cluster 2 co-citation literature. For example, the references about the role of macrophages during ALI in recruiting other immune cells, especially “neutrophils,” were widely cited. Also, references on inflammatory “cytokines” secreted by macrophages leading to “inflammation” in ALI were the main cited papers. In addition, although the keyword “polarization” had relatively few co-cited times, it was in the middle of the map, indicating that this topic was closely related to macrophages and ALI and needs further mining.

### Analysis of Research Hotspots

**Figure 5:**
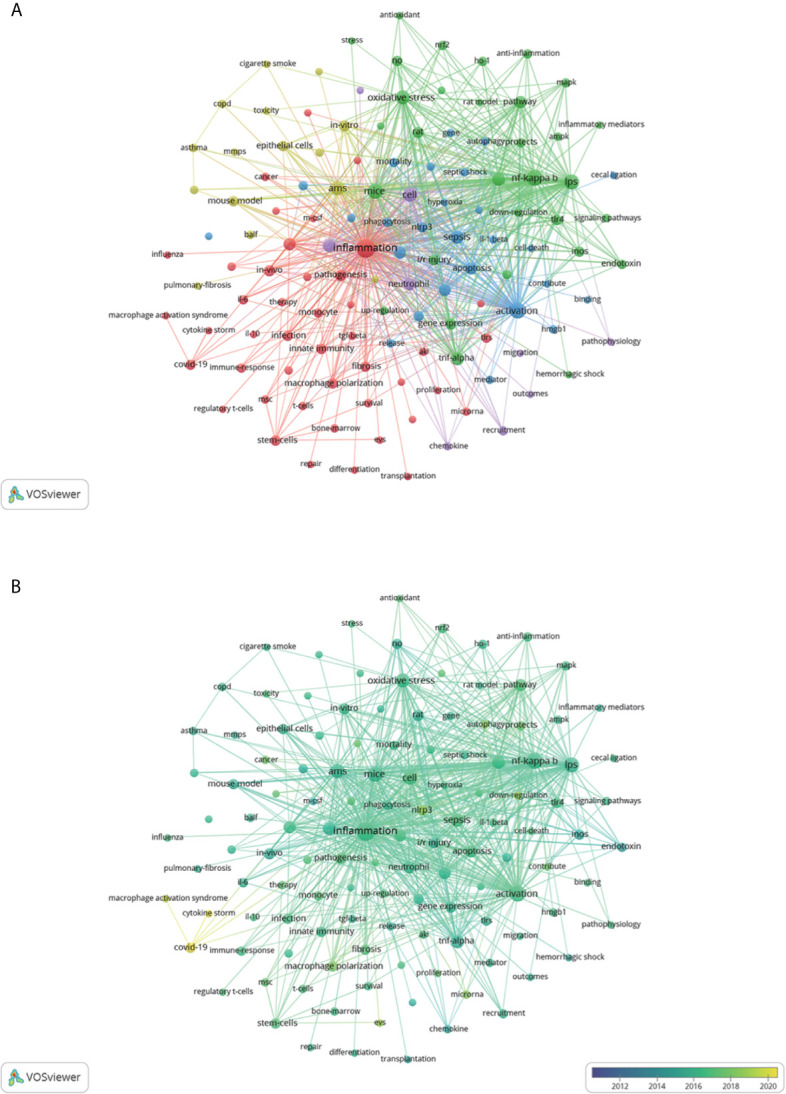
The mapping on keywords of macrophages in ALI. **(A)** The 125 keywords that occurred more than 25 times were divided into five clusters by different colors: cluster 1: red, cluster 2: green, cluster 3: blue, cluster 4: yellow, cluster 5: purple. The size of the nodes represents the frequency of occurrences. **(B)** Visualization of keywords according to the APY. Keywords in yellow appeared later than that in blue.

Apart from search terms, keywords extracted from the titles and abstracts of 2,632 papers were analyzed by VOSviewer (**Figure 5**). According to **Figure 5A**, cluster 1 was mainly about the role of macrophages in different stages of ALI. Cluster 2 and cluster 5 mainly reflected cellular mechanisms and molecular pathways of macrophage response to ALI. Cluster 3 focused on the function of macrophages in ALI induced by sepsis. Cluster 4 was mainly about the ALI caused by other diseases. The top frequent occurrences of keywords were “inflammation,” “NF- κB,” “activation,” “LPS,” and “mice,” suggesting that the researches related to macrophages in ALI mainly focused on basic studies. As shown in **Figure 5B**, the colors of all keywords were divided by VOSviewer according to the average publication year (APY). The latest keyword was “COVID-19” (cluster 1, APY: 2019.88), followed by “cytokine storm” (cluster 1, APY: 2019.67) and “macrophage activation syndrome” (cluster 1, APY:2019.15) both closely associated with COVID-19. Besides, “extracellular vesicles” (cluster 1, APY: 2018.39), and “NLRP3” (cluster 3, APY: 2018.14) were the recent major topics in this field (**Supplementary Table 2**). Compared with **Figures 5A**, **B**, the study of reducing inflammation to alleviate ALI was relatively the latest.

### Analysis of Research for Macrophages in ALI Caused by COVID-19

**Figure 6:**
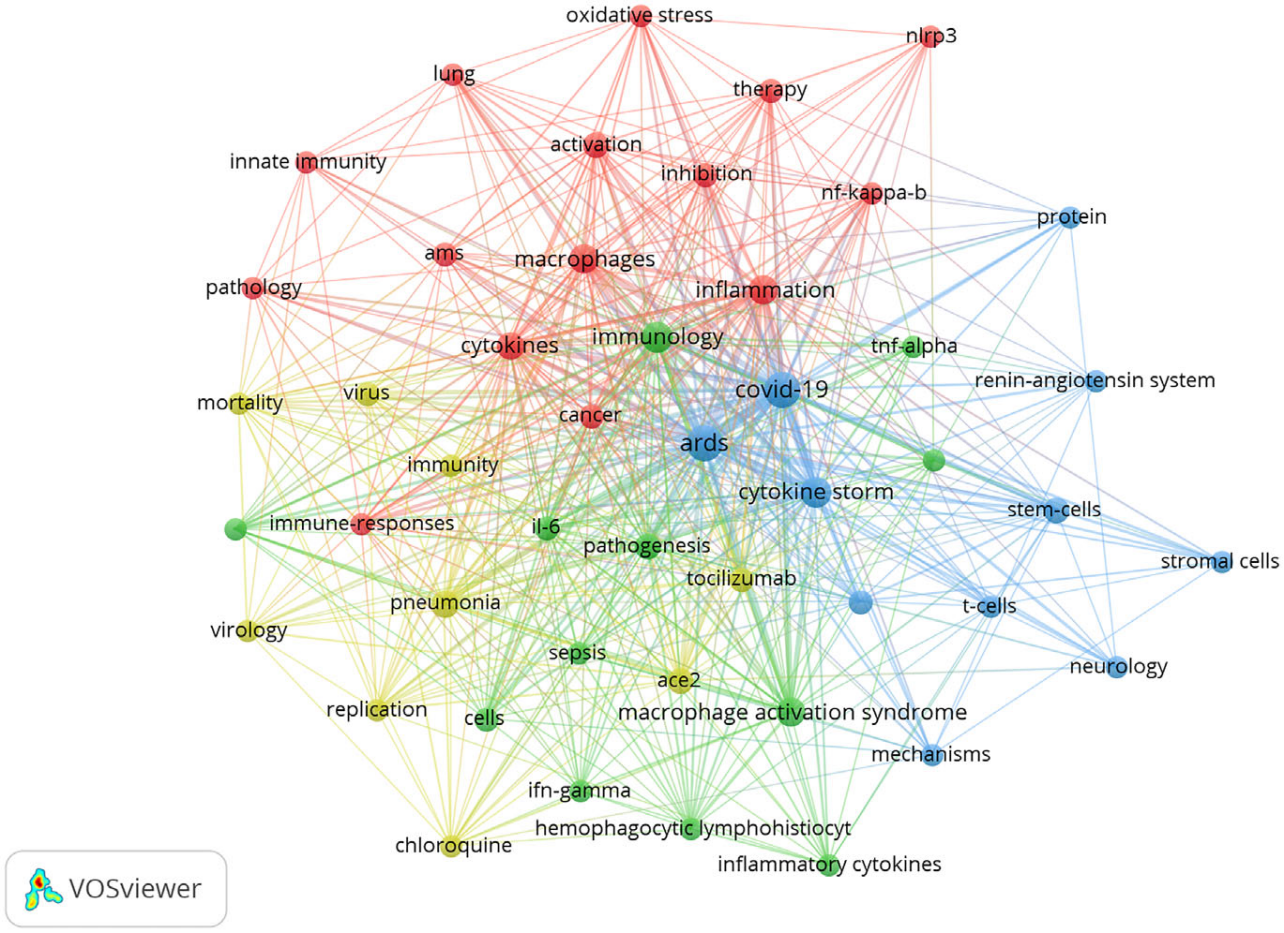
The network mapping on keywords of macrophages in ALI caused by COVID-19. The 47 keywords that occurred more than 4 times were divided into four clusters by different colors: cluster 1: red, cluster 2: green, cluster 3: blue, cluster 4: yellow. The size of the nodes represents the frequency of occurrences.

Under the epidemic of COVID-19, Np related to macrophages in ALI in 2020 increased sharply (**Figure 2A**), which was also reflected in **Figure 4A**. From the 2,632 articles we searched, 110 papers about macrophages in ALI related to COVID-19 were screened and analyzed for further understanding the trend and hotpots about macrophages in ALI caused by COVID-19 (**Figure 6**). As was shown in **Figure 6**, except for COVID-19, ARDS, and macrophages, cytokine storm, immunology, and inflammation appeared most frequently. Also, **Figure 6** showed the involvement of macrophages in the pathogenesis of ALI induced by COVID-19. What is more, the mechanism of anti-inflammatory treatment and stem cell therapy for ALI induced by COVID-19 ALI by inhibiting macrophages activation was revealed (**Figure 6**). In particular, there was considerable overlap between **Figure 6** and **Figure 5A**, suggesting that previous studies on macrophages in ALI were similar to those induced by COVID-19.

## Discussion

In this study, we conducted a bibliometric analysis to investigate the developmental trends and hotspots of researches on macrophages in ALI from the SCI-expanded database by using VOSviewer and Bibliometrix software. We retrieved 2,632 original articles and reviews published from 2011 to 2020. According to the polynomial-fitting curve, the annual number of publications showed an overall upward trend, rocketing up in the latter half of the period, especially after 2015. The ground-breaking publications with high LCS were the main reason for the rapid growth of the annual Np.

Among the top countries/regions, China ranked first in Np, suggesting that China was a highly productive country on macrophages in ALI. Seven Chinese affiliations and four Chinese authors came in the top 10 affiliations and authors in the research of macrophage researches in ALI, symbolizing that China possessed the most elite institutions and professional researchers around the world, which partially explained why China developed rapidly in this field over the past decade. However, compared with China, the USA had a relatively high Nc and H-index. This was because the important function of pulmonary alveolar macrophages in the inflammation and resolution of ALI was initially proposed by American scholars (28) and the USA went deeper in this field as compared with the rest of the world. This indicates that scholars and affiliations in China should make more efforts on the quality of their papers in this field. Similarly, the contradiction between the quantity and quality of publications also existed in South Korea.

Notably, of the top productive 10 journals, seven had high IF. This means that publishing research on macrophages in ALI in high-quality journals is not a challenge. Among the top 10 articles with high LCS, six were published in these high-IF journals, indicating that these journals had published a greater number of potential breakthroughs in this field, which reminded scholars interested in this topic to pay more attention to these journals.

As shown in the analysis of LCS, co-cited references and keywords, most researches in this field were basic studies, which needed animal models of ALI. Matute-Bello, G’s paper about these animal models had the highest LCS, meaning that his work was widely accepted by other researchers. This paper identified ALI in animals and determined the ALI characteristics of experimental animals (20). Besides, a review mentioned that sepsis was a common cause of ALI (29). As an important mediator of sepsis, lipopolysaccharide (LPS) activated macrophages *via* toll-like receptor 4 (TLR4) pathways in ALI. Meanwhile, the results of LPS are easy to reproduce within experiments. Therefore, LPS becomes a common substance used to establish a model of ALI (30). Based on these studies, animal models of ALI proposed various researches of pathogenesis about macrophages in ALI.

**Figure 5A** showed that the inflammation caused by excessive activation of macrophages in ALI was always the hot focus of research. Activated M1 secretes various inflammatory cytokines including TNF-α, IL-1β, IL-6, chemokines, and inducible nitric oxide synthase (iNOS), which may initiate ALI. M2 macrophages were found to be key orchestrators of the inflammatory resolution and repair in ALI, by producing a large number of anti-inflammatory factors such as IL-10 and TGF-β (4, 22). Recently, most researchers in this field focused on the complex function of two main phenotypic macrophage polarization in inflammatory, reparative, and fibrotic responses of ALI.

With the continuous progress of the research, the signal pathway involved in various mechanisms had been discovered. The mapping on keywords revealed that nuclear transcription factor (NF)-κB played a critical role in the pathogenesis of macrophages mediating ALI. As an important regulating factor, NF-κB was reported to promote the expression of M1 polarization-related genes (31), and inhibition of NF- κB could inhibit the polarization of M1, thus reducing the production of inflammatory cytokines and ALI (32). Meanwhile, oxidative stress products could directly trigger cytokine production in macrophages *via* the pathway of Toll-like Receptor 4 (TLR4) and NF-κB to modulate the severity of ALI (33). In addition, consistent with the results of LCS analysis, (NOD)-like receptor protein 3 (NLRP3) was the latest keyword in cluster 2, meaning that it was a relatively novel mechanism. L. Hou found that NLRP3 activated by various dangerous states (including infection and metabolic abnormalities) induced macrophage pyroptosis to release inflammatory mediators, resulting in ALI (34).

A comparison between in **Figure 5A** with **Figure 5B** revealed that researches on the protection of stem cells regulating macrophages to inhibit and reduce inflammation (cluster 4) have become a hotspot over the last 5 years. Gupta et al. discovered that treatment with intrapulmonary mesenchymal stem cells (MSC) decreased the severity of endotoxin-induced ALI and improved the survival rate in mice remarkably (35). However, the major treatment of stem cells in animals of ALI was mediated by extracellular vesicles (EVs), such as exosomes, microvesicles, and apoptotic bodies (36). Deng et al. found that bone marrow mesenchymal stem cells (BMSCs)-derived exosomes could inhibit M1 polarization and promote M2 polarization in sepsis-induced ALI by inhibiting glycolysis in macrophages (37). Additionally, MSC–derived EVs could target NF-κB to promote M2 polarization mitigating ALI *via* transferring microRNA (38). Furthermore, EVs which are low immunogenicity and easy to preserve (39), may become a substitute for stem cells to treat ALI.

Due to the outbreak of COVID-19 leading to ARDS in 2019, the Np of this field in 2020 reached the highest level, which was verified by cluster 6 in **Figure 4**. Meanwhile, according to **Figure 5**, the “cytokine storm” caused by alveolar macrophages was one of the main pathogenesis of COVID-19–induced ARDS. Also, histological investigation of bodies of two deceased cases with COVID-19 showed that abnormal macrophages infected by the virus promoted the damage of cytokine storm to lung tissue (40). Therefore, inhibiting the cytokine storm caused by macrophage activation may be one of the ways to treat COVID-19 related ALI. For example, studies have shown that stem cells could regulate the activation and polarization of macrophages, thus reducing pro-inflammatory cytokine to alleviate ALI (41, 42). At present, some COVID-19 clinical trials using stem cells are ongoing and most of them have shown benefits (43). Because of the complex pathogenesis about ALI related to COVID-19, no explicit and effective treatments are used clinically at present. In the light of the overlap between **Figure 5A** and **Figure 6**, the analysis of hotspots in the research about macrophages in ALI may help to clarify the mechanism of COVID-19–induced ARDS and search for potential treatments to ease the tension of global medical care caused by the lack of effective treatment for COVID-19.

Based on the bibliometrics analysis and visualization of the literature, to some extent, we can know the development trend and the hotspots in this field. At the same time, our study may help understand the important nodes in the trend of this field better by using LCS as an index. However, this study has some limitations. Firstly, only articles and reviews written in English from SCI-expanded were included. Secondly, the VOSviewer may omit some information because it is unable to analyze the full texts of the publications. Lastly, this study has a hysteretic quality to some degree due to exclusion of some newly published outstanding papers with low Nc.

## Conclusion

This analysis of bibliometric reveals that researches on macrophages in ALI are developing rapidly at present. China is a major producing country, and the USA has made many outstanding breakthroughs in this field. AM J PHYSIOL-LUNG C and AM J RESP CELL MOL have published the latest studies and novel progress in this field. Recently, the role of molecular pathways of NLRP3 and EVs in attenuating ALI by regulating macrophages has become a hotspot of researches. Moreover, COVID-19–induced ALI may be associated with cytokine storms caused by macrophage activation.

## Data Availability Statement

The original contributions presented in the study are included in the article/**Supplementary Material**. Further inquiries can be directed to the corresponding authors.

## Author Contributions

SW, HZ, and LZhe did this bibliomrtrics analysis and wrote manuscript. WZ, LZhu, DF, JW, GC, XJ, and HY participated in experimental design and manuscript writing. XS and XL designed this study and organized the manuscript writing. All authors contributed to the article and approved the submitted version.

## Funding

Funding was received from National Natural Science Foundation of China (no. 81871601) Development Fund for the Department of Anesthesiology, Shanghai Pulmonary Hospital.

## Conflict of Interest

The authors declare that the research was conducted in the absence of any commercial or financial relationships that could be construed as a potential conflict of interest.
