# Supplementary material for: Global Trends in Research of Macrophages Associated With Acute Lung Injury Over Past 10 Years: A Bibliometric Analysis
Source: Front Immunol. 2021 May 20;12:669539. doi: 10.3389/fimmu.2021.669539 (PMC8173163; doi:10.3389/fimmu.2021.669539)
Supplement: Supplementary file 1 [file DataSheet_1.pdf]

S table 1. The 31 references of cluster 2 in Figure 4A

	Athour	DOI	Citations	Title
1	aggarwal nr	10.1152/ajplung.00341.2013	106	Diverse macrophage populations mediate acute lung inflammation and resolution
2	herold s	10.3389/fimmu.2011.00065	70	Acute lung injury: how macrophages orchestrate resolution of inflammation and tissue repair
3	johnston lk	10.1165/rcmb.2012-0090oc	65	Pulmonary macrophage subpopulations in the induction and resolution of acute lung injury
4	mosser dm	10.1038/nri2448	62	Exploring the full spectrum of macrophage activation
5	hussell t	10.1038/nri3600	52	Alveolar macrophages: plasticity in a tissue-specific context
6	sica a	10.1172/jci59643	48	Macrophage plasticity and polarization: in vivo veritas
7	d'alessio fr	10.1172/jci36498	46	CD4+CD25+Foxp3+ Tregs resolve experimental lung injury in mice and are present in humans with acute lung injury
8	gordon s	10.1016/j.immuni.2010.05.007	45	Alternative activation of macrophages: mechanism and functions
9	janssen wj	10.1164/rccm.201011-1891oc	44	Fas determines differential fates of resident and recruited macrophages during resolution of acute lung injury
10	gordon s	10.1038/nri978	42	Alternative activation of macrophages
11	misharin av	10.1165/rcmb.2013-0086ma	41	Flow cytometric analysis of macrophages and dendritic cell subsets in the mouse lung
12	murray pj	10.1016/j.immuni.2014.06.008	41	Macrophage activation and polarization: nomenclature and experimental guidelines
13	frank ja	10.1152/ajplung.00055.2006	38	Alveolar macrophages contribute to alveolar barrier dysfunction in ventilator-induced lung injury
14	murray pj	10.1038/nri3073	36	Protective and pathogenic functions of macrophage subsets
15	rosseau s	10.1152/ajplung.2000.279.1.L25.	35	Phenotypic characterization of alveolar monocyte recruitment in acute respiratory distress syndrome
16	martinez fo	10.1146/annurev.immunol.021908.132532	34	Alternative activation of macrophages: an immunologic functional perspective
17	gordon s	10.1038/nri1733	33	Monocyte and macrophage heterogeneity
18	jiang dh	10.1038/nm1315	31	Regulation of lung injury and repair by Toll-like receptors and hyaluronan
19	beck-schimmer b	10.1186/1465-9921-6-61	30	Alveolar macrophages regulate neutrophil recruitment in endotoxin-induced lung injury
20	vergadi e	10.4049/jimmunol.1300959	30	Akt2 deficiency protects from acute lung injury via alternative macrophage activation and miR-146a induction in mice
21	hashimoto d	10.1016/j.immuni.2013.04.004	29	Tissue-resident macrophages self-maintain locally throughout adult life with minimal contribution from circulating monocytes
22	gibbons ma	10.1164/rccm.201010-1719oc	28	Ly6Chi monocytes direct alternatively activated profibrotic macrophage regulation of lung fibrosis
23	mantovani a	10.1016/j.it.2004.09.015	28	The chemokine system in diverse forms of macrophage activation and polarization
24	zhao mq	10.1152/ajplung.00086.2006	28	Alveolar macrophage activation is a key initiation signal for acute lung ischemia-reperfusion injury

25	dhaliwal k	10.1164/rccm.201112-2132oc	26	Monocytes control second-phase neutrophil emigration in established lipopolysaccharide-induced murine lung injury
26	guilliams m	10.1084/jem.20131199	26	Alveolar macrophages develop from fetal monocytes that differentiate into long-lived cells in the first week of life via GM-CSF
27	lawrence t	10.1038/nri3088	26	Transcriptional regulation of macrophage polarization: enabling diversity with identity
28	fadok va	10.1172/jci1112	25	Macrophages that have ingested apoptotic cells in vitro inhibit proinflammatory cytokine production through autocrine/paracrine mechanisms involving TGF-beta, PGE2, and PAF
29	laskin dl	10.1146/annurev.pharmtox.010909.105812	25	Macrophages and tissue injury: agents of defense or destruction?
30	westphalen k	10.1038/nature12902	25	Sessile alveolar macrophages communicate with alveolar epithelium to modulate immunity
31	wynn ta	10.1038/nature12034	25	Macrophage biology in development, homeostasis and disease

S table 2. The top 10 occurrences and the top 10 new APY keywords in Figure 5

	Keyword	Occurrences	Cluster	Keyword	APY	cluster
1	inflammation	1042	1	covid-19	2019.88	1
2	nf-kappa b	573	2	cytokine storm	2019.67	1
3	activation	515	3	macrophage activation syndrome	2019.15	1
4	lps	463	2	evs	2018.39	1
5	mice	374	2	nlrp3	2018.14	3
6	cell	333	5	down-regulation	2018.1	2
7	ams	326	4	autophagy	2017.98	3
8	oxidative stress	287	2	microrna	2017.95	1
9	sepsis	285	3	macrophage polarization	2017.8	1
10	inhibition	257	2	contribute	2017.57	3
